# Risk factors for critical forms of SARS-CoV-2 infection in fully vaccinated patients: a prospective observational study

**DOI:** 10.11604/pamj.2022.43.124.32265

**Published:** 2022-11-04

**Authors:** Yassine Bou-ouhrich, Boubaker Charra

**Affiliations:** 1 Department of Medical Intensive Care, Ibn Rochd University Hospital, Faculty of Medicine and Pharmacy of Casablanca, Hassan 2 University, Casablanca, Morocco

**Keywords:** COVID-19 vaccination, SARS-CoV-2 infection, resuscitation

## Abstract

The novel coronavirus disease of 2019 (COVID-19) vaccination is a critical prevention measure to help sort out the COVID-19 crisis. Nonetheless, since the beginning of the pandemic, it has been well-documented that older adults as well as people enduring an immunocompromised condition are the most likely to develop a severe COVID-19 form owing to a less robust immune system and therefore a weaker immunologic response to COVID-19 vaccination. Herein, we report an observational prospective monocentric study of a series made up of 30 patients fully vaccinated against Severe Acute Respiratory Syndrome-Coronavirus-2 (SARS-CoV-2) out of a total of 139 patients admitted to the medical intensive care unit (ICU) for a critical form of COVID-19 between February 2021 and October 2021. This observational study was conducted during the peak of the pandemic outbreak and therefore its main aim was to describe the epidemiological and sociodemographic features of fully vaccinated patients who endured critical forms of COVID-19. Immunocompromised people as well as those with chronic underlying comorbidities are more likely to develop critical forms of COVID-19. Moreover, it seems that vaccine efficacy decreases gradually over time. SARS-CoV-2 variants may also undermine vaccine effectiveness. Supplemental doses would be of paramount in higher-risk people to build on protective immunity against COVID-19. Further randomized controlled trials are also desperately needed to determine the optimal interval between primary series and booster doses of the several COVID-19 vaccines chiefly for the vulnerable people.

## Introduction

The Coronavirus disease-2019 (COVID-19) pandemic, caused by the Severe Acute Respiratory Syndrome-Coronavirus-2 (SARS-CoV-2), has been a significant global health threat for the past couple of years [1]. Several risk factors have been suggested for COVID-19 infection such as older age, presence of comorbidities, and prior pneumonia [2,3]. Due to the high morbidity and mortality of COVID-19 [4,5], along with several quarantine regulations, the importance of vaccination is being emphasized. Following the demonstration of effective prevention of COVID-19 by several vaccines in phase-3 trials, they have been approved [6-9]. However, even fully vaccinated people remain at risk of developing critical forms of COVID-19 [10,11]. Causes of breakthrough infections in vaccinated patients include incomplete vaccine efficacy, decline in protective effects over time, differences in individual immune responses after vaccination, and emergence of variants [12]. With increases in population vaccine coverage, the number of breakthrough infection cases is expected to rise [13]. To date, little is known about how prior vaccination affects the clinical outcomes and prognosis of breakthrough infections in fully vaccinated patients. The aim of this observational study is to describe the epidemiological and sociodemographic characteristics that might potentially affect the overall prognosis of fully vaccinated COVID-19 patients.

## Methods

**Study design and setting**: the study has been designed as an observational hospital-based prospective monocentric study carried out in the medical intensive care unit of the Ibn Rochd University Hospital of Casablanca consisting of a series of 30 patients fully vaccinated against SARS-CoV-2 out of a total of 139 patients admitted to the unit from 01/02/2021 to 07/10/2021 for a critical form of COVID-19.

**Study participants and recruitment**: recruitment of participants was based upon inclusion and exclusion criteria among all patients admitted to the medical intensive care unit. Inclusion criteria included vaccinated patients (admitted after 14 days of the 2^nd^ vaccine dose), positive COVID-19 real time-polymerase chain reaction (RT-PCR), patients hospitalized for a critical form of COVID-19. However, non-vaccinated patients and patients who received only one primary dose of vaccine were excluded. A critical form of COVID-19 has been operationally defined by the national expert committee by the presence of at least one of the following criteria: 1) respiratory rate ≥30 cycles per minute; 2) pulse oxygen saturation (SPO2) < 92% with ≥4 liters/min of oxygen; 3) altered mental state; 4) systolic blood pressure < 90 mmHg; 5) need of vasopressors; 6) serum lactate level > 2mmol/l; 7) qSOFA (quick sequential organ failure assessment) ≥2 points.

**Data collection and analysis**: clinical and paraclinical data were collected and measured using bedside monitoring and laboratory tests. Quantitative and qualitative variables as well as subgroups interactions have been handled and analyzed using Excel software and presented with percentages and figures. Data entry was done using Excel software, and analysis was performed using the 16 SPSS version software. A univariate descriptive analysis was carried out where proportions, numbers, means, and standard deviations (S.D) were calculated. A p-value ≤ 0.05 was considered significant.

**Ethics approval statement**: the Research Ethics Committee has confirmed that no ethical approval is required for a purely observational study.

## Results

**Figure 1:**
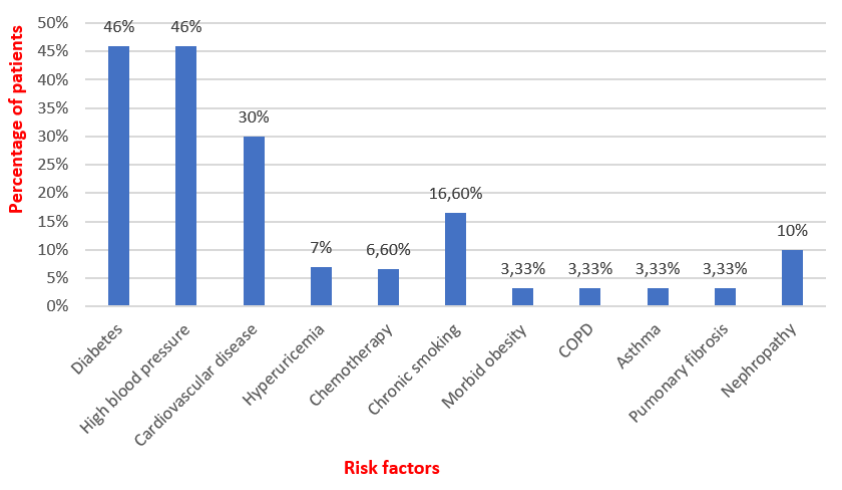
bar chart displaying main risk factors found in patients of our series

**Figure 2:**
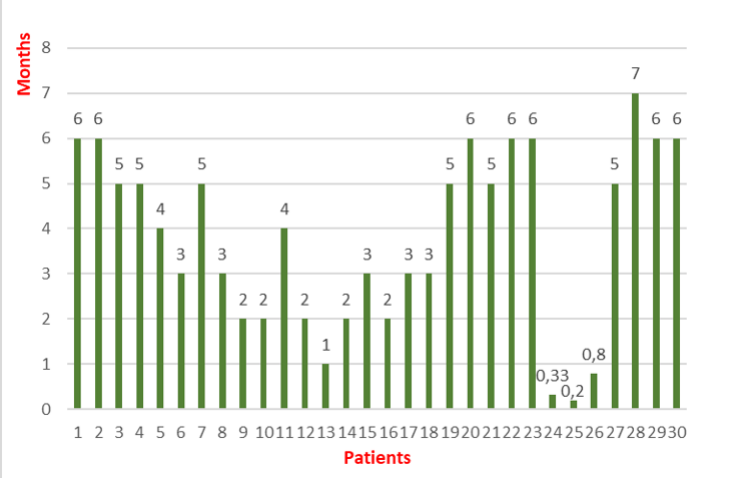
bar chart showing the interval between the second jab and the onset of symptoms

**Baseline characteristics**: thirty patients fully vaccinated against COVID-19 were admitted to the medical intensive care unit of the Ibn Rochd University Hospital in Casablanca for critical forms of SARS-CoV-2 pneumonia. The responsible variants were not determined but the Delta variant was the most prevalent in Morocco during the study period. Twenty-one patients, 70%, were male and nine patients, 30%, were female. The mean age was 69 years old with extremes ranging from 48 to 89 years old. Fourteen patients, roughly 46%, had type 2 diabetes evolving for more than five years in 23% of cases and for more than ten years in 23% of cases. Fourteen patients, around 46%, had high blood pressure that had been evolving for more than five years in 23% of cases and for more than ten years in 23% of cases. Nine patients, 30%, had been being followed for a cardiovascular disease with ischemic cardiomyopathy in 24% of cases, complete arrhythmia by atrial fibrillation in 3% of cases, and aneurysm of the ascending aorta in 3% of cases. Three patients, around 10%, had been being followed for chronic nephropathy. Two patients, nearly 6.60%, had been taking allopurinol for hyperuricemia, and two other patients, 6.60%, had been undergoing chemotherapy for colon cancer and large B-cell lymphoma. Five patients, approximately 16.60%, were chronic smokers. One patient was morbidly obese. One patient had been being followed for chronic obstructive pulmonary disease and another patient for asthma. One patient was under long-term corticosteroid therapy for pulmonary fibrosis (Figure 1). Eighteen patients in our series, around 60%, were vaccinated with an adenovirus vector vaccine while twelve patients, around 40%, received an inactivated virus vaccine. The average time between the second vaccine dose and the onset of clinical symptoms was roughly four months with extremes spanning from twenty days up to seven months (Figure 2).

**Clinical presentation**: on admission to the intensive care unit, twenty-two patients, just under 75%, were conscious with a Glasgow coma scale of 15/15 while six patients, about 20%, were confused. The average pulse oxygen saturation was 72% in room air ranging from 40% to 92%. The mean respiratory rate was 27 cycles per minute varying between 12 and 45 cycles per minute. All patients were, however, hemodynamically stable and apyretic.

**Paraclinical features**: the chest computed tomography scan showed interstitial damage interesting more than 50% of the lung parenchyma in twenty-four patients, around 80%, with extremes ranging from 5% to 98% of lung involvement. Biologically, the average C-reactive protein (CRP) level was 179.70 mg/l spanning from 26.7 to 391.8 mg/l. Twenty-two patients, just over 70%, had a neutrophilic hyperleukocytosis with a maximum of 30230/mm^3^. Twenty-four patients, about 80%, had an average lymphopenia of 990/mm^3^ varying from 90 to 3300/mm^3^. The procalcitonin (PCT) serum level was elevated in eight patients, 26%. The mean fibrinogenemia was 5.96 g/L with extremes ranging from 3.11 to 9.70 g/L. The average D-dimer level was 4293 µg/l spanning from 100 to 38050 µg/l. The rest of the hemostasis work-up, in particular the prothrombin level and the activated cephalin time, showed no abnormalities in all patients.

**Figure 3:**
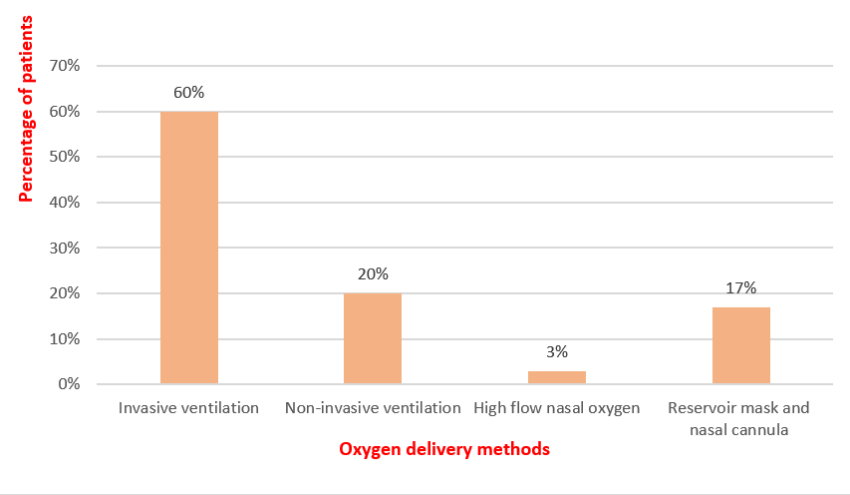
pie chart showing oxygen delivery methods in our patients

**Management**: with regard to therapies, eighteen patients, 60%, needed invasive ventilation, six patients, 20%, required non-invasive ventilation, one patient was put under high-flow nasal oxygen therapy, and five patients, about 16.66%, responded well to non-rebreather mask and nasal oxygen cannula (Figure 3). Empirical antibiotic therapy, which was subsequently adapted to antibiogram, was administered to all patients. Several Antibiotics were prescribed such as third generation cephalosporins in particular ceftriaxone and ceftazidime; Imipenem; Amikacin; Gentamicin; Ciprofloxacin; Moxifloxacin; Vancomycin, Teicoplanin, Piperacillin/Tazobactam. Thromboprophylaxis was assured by curative low molecular weight heparin therapy in twenty-seven patients, 90%, and by standard heparin in three patients, 10%. Corticosteroid therapy based on 80mg per day of methylprednisolone over 10 days as well as acetylsalicylic acid as an antiaggregant agent were also administered to all patients. Tocilizumab was, however, administered to eight patients, about 26%.

**Figure 4:**
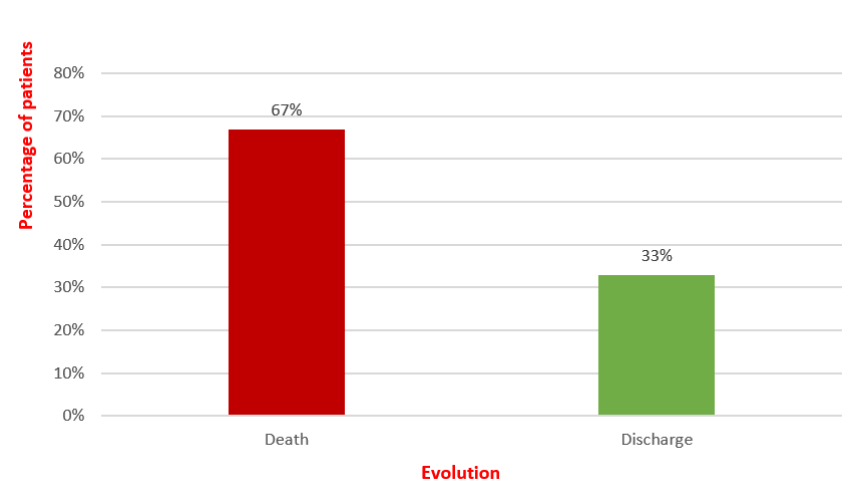
pie chart showing the evolution of patients

**Evolution**: around twenty patients, roughly 66%, had poor outcomes (Figure 4), and death occurred on average ten days after ICU admission.

## Discussion

The immunogenicity of COVID-19 vaccines has been demonstrated to be effective up to 6-8 months following vaccination [14]. Two studies have actually shown lower neutralization titers over the 2-3 months post-vaccination of the adenoviral vector vaccine compared with messenger ribonucleic acid (mRNA) vaccines including against Beta and Delta variants [15]. Another recent modeling study, based on immunogenicity data, predicted that vaccine efficacy against symptomatic infection caused by the Delta variant may drop to less than 50% during the first year after vaccination for the majority of vaccines currently in use globally. Nevertheless, the majority remain protected against critical forms of COVID-19 [16]. Indeed, in spite of an overall clinical efficacy at six months of 91-97% of the mRNA vaccine against severe coronavirus disease, a non-significant decrease of six percentage points was observed every two months after seven days post-vaccination: 96% between seven days and two months, 90% between two and four months, and 84% between four and six months [17]. The noticed changes in vaccine effectiveness may therefore reflect a decline in vaccine performance against the Delta variant, a decrease in the immunity acquired by the primary vaccination, or other unmeasured confounders. Furthermore, as vulnerable people were generally vaccinated first, observational studies analyzing the duration of protective immunity might be biased by risk status. A retrospective cohort study in a large healthcare system in Israel noted a 2.3-fold increased risk for infection among fully vaccinated persons who were vaccinated with mRNA vaccine in January vs. April 2021 [18]. A similar study observed a higher rate (2.4% vs 1.1%, OR=2.2) of infection in fully vaccinated persons who received the second mRNA dose more than five months ago compared with those who received it less than five months ago, with higher magnitude of difference with increasing age [19]. Another point to stress is that cellular immunity may actually help to limit disease severity in infections caused by variants that partially escape neutralizing antibodies. Variations in genes encoding human leukocyte antigens have also been observed resulting thus in variation regarding T cell response to specific SARS-CoV-2 variants, this may have a different impact on the population depending on the genetic prevalence of these variations [20]. There are currently no studies of vaccine-induced cellular immunity against the Delta variant.

Actually, among hospitalized or fatal cases reported to the center for disease control (CDC) as of August 30, 2021, 70% of hospitalized and 87% of fatal COVID-19 cases in fully vaccinated people were in persons aged 65 years and older owing to lower antibody levels compared with those with sustained protection, as shown in a study of fully vaccinated health care workers in Israel with Delta variant infections [10]. Other studies in the United States and Israel have also found that immunocompromised people account for a high proportion (≥40%) of infections among fully vaccinated hospitalized patients [21]. Reduced antibody response to a two-dose primary series of mRNA COVID-19 vaccines has also been noticed in specific groups of immunocompromised adults, including people receiving solid organ transplants [22]; some people with cancer, particularly hematologic cancers [23]; some people receiving hemodialysis for kidney failure [24]; and those taking immunosuppressive agents [25]. Therefore, emerging data suggest that an additional dose in immunocompromised individuals, typically given at least 28 days after completion of the primary series, would strengthen the humoral response since in small observational studies of solid organ transplant recipients [26] and chronically hemodialyzed patients [27], 33-54% of those who had no detectable antibody response after the initial double-dose series of mRNA vaccine developed a humoral response to the supplemental dose. Moreover, the Delta variant, first detected in India, has been shown to have increased transmissibility, potentially weakened neutralization by monoclonal antibodies, as well as reduced neutralization by post-vaccination sera. A study in Houston, Texas, noted that Delta caused a significantly higher rate of infection in fully vaccinated individuals compared with other variants, but noted as well that only 6.5% of all COVID-19 cases occurred in fully vaccinated people [28]; similar findings have been observed in India. Indeed, early clinical data indicate that vaccinated and unvaccinated patients infected with the Delta variant have similar levels of viral ribonucleic acid (RNA) and detectable culturable virus, meaning that some fully vaccinated people infected with the Delta variant may be contagious [29].

**Limitations of the study**: 1) the polymedication and the association with other clinical conditions like sepsis, which would also have affected the overall prognosis, made it difficult to individualize the effect of vaccination in severe COVID-19 cases. 2) Limited sample size. 3) Missing data have not been included in the investigation which may have exposed to attrition bias. 4) The study is monocentric which might undermine its generalisability.

## Conclusion

Vaccination is certainly an effective way to help tackle COVID-19 pandemic and bring down mortality. Supplemental doses are therefore desperately needed for vulnerable people, both young and older, to underpin the immunologic response against SARS-CoV-2 infection. Social distancing and barrier gestures remain mandatory as well in order to limit the community transmission of the virus.

### What is known about this topic

- Vaccine efficacy decreases gradually over time;
- Immunocompromised people are more likely to develop a critical form of COVID-19.

### What this study adds

- Vaccination is the most reliable way to protect vulnerable people from critical COVID-19;
- Two-dose primary series COVID-19 vaccine are not sufficient to build on a strong protective immunity especially with the ongoing SARS-CoV-2 iterations;
- Vaccine efficacy against COVID-19 is influenced by the interval between the booster doses.
